# Investigation of the Origin of High Photoluminescence Quantum Yield in Thienyl-S,S-dioxide AIEgens Oligomers by Temperature Dependent Optical Spectroscopy

**DOI:** 10.3390/molecules28135161

**Published:** 2023-07-01

**Authors:** Marco Anni

**Affiliations:** Dipartimento di Matematica e Fisica “Ennio De Giorgi”, Università del Salento, Via per Arnesano, 73100 Lecce, Italy; marco.anni@unisalento.it

**Keywords:** AIE molecules, conjugated molecules, oligothiophene-S,S-dioxides, organic electronics, photoluminescence, time resolved spectroscopy

## Abstract

The development of organic molecules showing high photoluminescence quantum yield (PLQY) in solid state is a fundamental step for the implementation of efficient light emitting devices. In this work the origin of the high PLQY of two trimers and two pentamers having one central thiophene-S,S-dioxide unit and two and four lateral thiophene or phenyl groups, respectively, is investigated by temperature dependent photoluminescence and time resolved photoluminescence measurements. The experimental results demonstrate that the molecules with lateral phenyl rings show higher PLQY due to a weaker coupling with intramolecular vibrations—related to variations in the radiative and non-radiative decay rates—and indicate different molecular rigidity as the main factors affecting the PLQY of this class of molecules.

## 1. Introduction

Organic conjugated molecules, both oligomers and polymers, are receiving large research attention in the last decades, both for fundamental and applicative interest. In particular, the capability to combine the typical active properties of semiconductors with the chemical flexibility and easy processability of plastic materials has opened the way to a wide range of possible applications of conjugated molecules, including solar cells [1], photodetectors [2], Field Effect Transistors (FETs) [3], Light Emitting Diodes (LEDs) [4], LASER [5,6], sensors [7,8,9], and biomarkers [10].

Limiting the attention to light emitting devices, such as LEDs and lasers, the active molecules are typically used in thin films, thus the ideal active material should show good emission properties in the solid state. Unfortunately, many organic molecules show better emission properties when isolated in dilute solutions than in the solid state, due to the appearance of aggregation related non-radiative decay channels [11,12,13] that decrease the photoluminescence quantum yield (PLQY) and limit the possible performance of the final devices.

Rather interestingly, some molecules show the opposite behaviour, with lower PLQY in solution than in the solid state (typically known as Aggregation Induced Emission luminogens, AIEgens), and are, thus, very interesting candidates as active materials in light emitting devices [14,15,16,17].

Within the different families of organic compounds, thiophenes are among the most studied, as they are chemically stable, easy to process, and naturally characterised by high aromaticity [18,19]. In addition, thiophenes are extremely versatile, thus allowing a wide tuning of their properties by acting on the molecular structure [20,21,22]. These properties, combined with good charge mobility, have allowed thiophenes applications in solar cells [23,24,25] and FETs [26,27,28]. On the contrary, the applications of standard thiophenes to light emitting devices is limited by their lower PLQY in solid films than in solution [29,30], mainly due to efficient cofacial packing of the molecular backbones and to sulphur-mediated strong Spin-Orbit coupling, leading to efficient Inter System Crossing (ISC) toward triplet states [18,31,32,33,34] and making the non-radiative decay channels dominant as compared to the radiative ones.

However, it has been demonstrated that the functionalisation of the sulphur atom of the central thiophene ring with two oxygen atoms allows one to strongly modify the electronic properties of the molecules, leading to wide color tunability [35,36], increased electron affinity [37], good solubility in common organic solvents with good film forming properties [38], and high chemical stability [39].

High PLQY has been obtained, both in solution and in the solid phase, in compounds containing rigid core oligothiophene-S,S-dioxides with bulky substituents, thanks to the combination of high molecular rigidity and reduced intermolecular interactions [40,41,42,43].

Even more interesting is the behaviour of oligothiophene-S,S-dioxides with flexible backbone, that, in marked contrast with the non functionalised molecules, show the typical AIEgens behaviour, with low PLQY in solution but very high PLQY (up to 70%) in the solid state [17,38,41,44,45,46]. In addition, high tunable optical gain has been demonstrated [47], thus making oligothiophene-S,S-dioxides excellent candidates as active materials for light emitting devices, such as LEDs and lasers [20,22,48,49,50,51].

Rather surprisingly, despite the wide experimental work on the improvement of the light emission properties of this class of compounds by acting on the molecular structure [20,22,51], all the works aiming to understand the origin of their emission properties have been, to date, performed only by optical spectroscopy experiments in solution, though sometimes complemented by theoretical calculations [35,41,43,46,52,53]. The conclusion of these works is that, in isolated oligothiophene-S,S-dioxides, the main non-radiative channel is no longer ISC, due to the reduction of the contribution of the central sulphur lone pair to Spin-Orbit coupling, but Internal Conversion (IC) [29,41,46,54]. The dominance of IC leads to PLQY in solution increasing with the solvent viscosity [35,41,55], which suggests that the high PLQY in the solid state could come from an extremely viscous environment.

However, despite this guess, to date, no direct experimental investigation of the basic photophysics of oligothiophene-S,S-dioxides in the solid state has been performed.

This work aims to fill this gap, by investigating the radiative and non-radiative decay processes in four functionalised oligothiophene-S,S-dioxides with different conjugation length (trimer vs. pentamer) and lateral ring functionalisation (thiophene vs. phenyl), by temperature dependent photoluminescence and time resolved photoluminescence measurements. The experimental results demonstrate that, at room temperature, the different functionalisation only affects the non-radiative decay rate, which is smaller in trimers than in pentamers and in molecules with lateral phenyl rings than the ones with thiophene rings, evidencing that the different PLQY (between 12% and 70%) is mainly due to differing importances of the non-radiative decay processes. It is also shown that the PL intensity decreases with temperature, with two thermally activated quenching processes, with typical activation energies of a few millielectronvolts to a few tens of millielectronvolts, related to lateral rings’ libration and intraring vibrations, respectively. The thermal activation of intramolecular vibrations induces not only an increase in the non-radiative decay rate, but also a decrease in the radiative decay rate, which is quantitatively dependent on the molecular structure. In particular, the molecules with lateral phenyl rings show higher PLQY than the corresponding oligothiophenes due to much lower coupling constants with the vibrations, evidencing a higher intramolecular rigidity.

Overall, these results demonstrate that the PLQY at room temperature is mainly determined by the intramolecular properties, and that the molecular rigidity, together with low intramolecular interactions, is the key parameter to minimise the non-radiative relaxation processes and to maximise the PLQY.

## 2. Results

**Figure 1:**
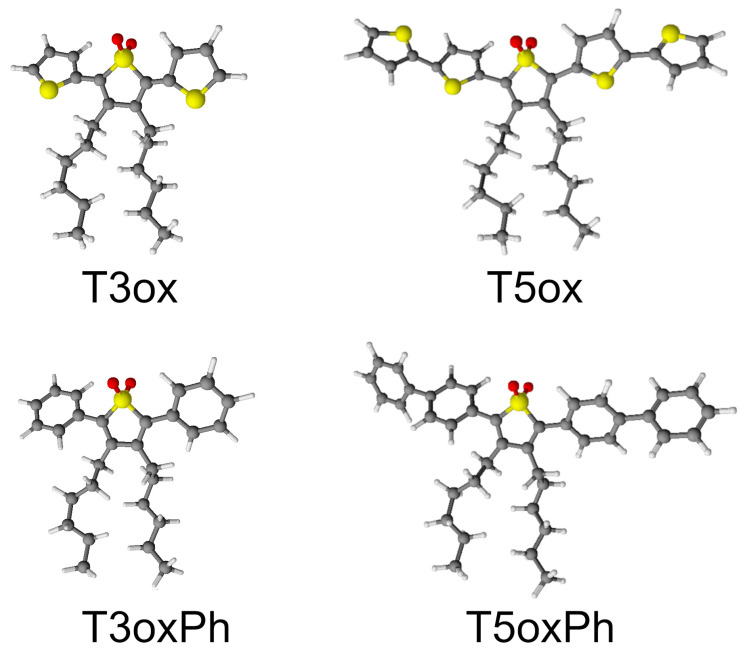
Chemical structure of the investigated molecules. The grey, yellow, red, and white dots represent C, S, O, and H atoms, respectively.

The chemical structure of the investigated molecules is reported in Figure 1. All the molecules have a common central thienyl-S,S-dioxide ring, with hexyl groups in the β position in order to make them soluble in common organic solvents (such as chloroform, dichloromethane, tetrahydrofuran (THF), toluene, and decalin) [41,49], but show different lateral rings and ring number. In particular, T3ox is a trimer with two lateral thiophene rings (3′,4′-dihexyl-[2,2′:5′,2${}^{\prime \prime }$-terthiophene] 1′,1′-dioxide), T3oxPh is a trimer with two lateral phenyl rings (3,4-dihexyl-2,5-diphenylthiophene 1,1-dioxide), T5ox is a pentamer with four lateral thiophene rings (3${}^{\prime \prime }$,4${}^{\prime \prime }$-dihexyl-[2,2′:5′,2${}^{\prime \prime }$:5${}^{\prime \prime }$,2${}^{\prime \prime \prime }$:5${}^{\prime \prime \prime }$,2${}^{⁗}$-quinquethiophene] 1${}^{\prime \prime }$,1${}^{\prime \prime }$-dioxide), and T5oxPh is a pentamer with four lateral phenyl rings (2,5-di([1,1′-biphenyl]-4-yl)-3,4-dihexylthiophene 1,1-dioxide). These molecules, thus, allow one to study the effects on the emission properties of the lateral ring chemical structure and of the molecule length.

**Figure 2:**
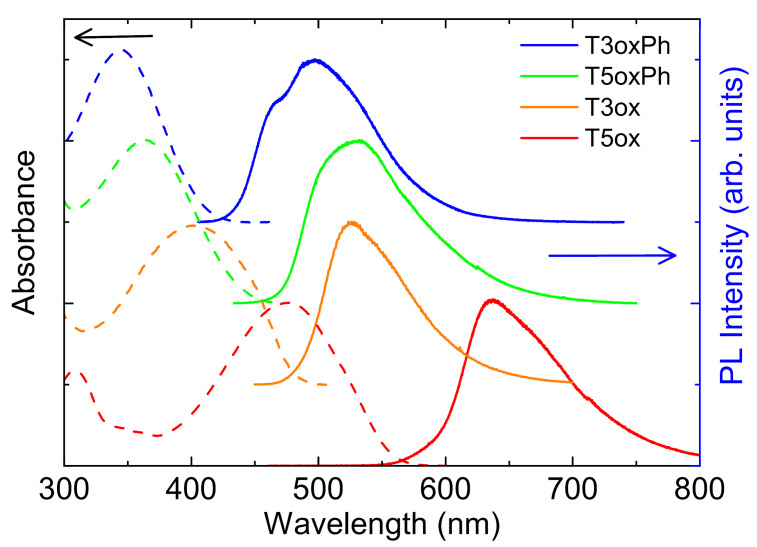
Absorbance and PL spectra of the investigated molecules. The spectra are normalised to 1 and vertically offset for clarity.

As a first step in the investigation of the optical properties of the molecules, their absorption and emission spectra and the PLQY were measured at room temperature. The spectra (see Figure 2) show a first absorption peak between the near-UV in T3oxPh and the blue–yellow in T5ox, and emission between cyan in T3oxPh and red in T5ox.

Looking at the effects of the molecular chemical structure, a blue-shift of the spectra of T3oxPh and T5oxPh, with respect to the corresponding T3ox and T5ox, is observed. In addition, both for the phenyl and thiophene lateral groups, the trimers show blue-shifted spectra with respect to the corresponding pentamer. These results indicate that, beyond the higher localisation in the shorter molecules with respect to the longer ones, the functionalisation with lateral phenyl rings leads to a higher exciton localisation along the molecule.

Concerning the PLQY values (see Table 1), the lowest value is observed in T5ox (12%), which is 6 times higher than the one in unsubstituted quinque-thiophene [56], with very high values in all the other molecules. In particular, the substitution of the external thiophene rings with the phenyl ones increases the PLQY up to 63% in T3oxPh and to 70% in T5oxPh.

In order to have a first insight into the origin of the high PLQY and of its clear increase in the phenylated molecules, the PL relaxation dynamics were investigated by time resolved PL measurements. The obtained results allow one to observe a bi-exponential decay in T3ox (discussed in further detail later) and a monoexponential decay in the other three molecules (see Figure 5).

The simultaneous knowledge of the PLQY and of the PL decay time (τ) allows one to separate the radiative ($\tau_{r}$) and non-radiative ($\tau_{nr}$) decay times by exploiting the following relations:

(1) $$\begin{array}{c} PLQY=\frac{\tau }{\tau_{r}} \end{array}$$

(2) $$\begin{array}{c} \frac{1}{\tau }=\frac{1}{\tau_{r}}+\frac{1}{\tau_{nr}} \end{array}$$

The obtained values (see Table 1) clearly show that the higher PLQY of the T3oxPh and T5oxPh, with respect to the T3ox and T5ox, can be fully ascribed to a strong increase in the non-radiative decay time (about 2 times in the trimers and 12 times in the pentamer), without appreciable differences in the radiative decay times. It is also interesting to observe that the four molecules have basically comparable radiative decay times, evidencing a comparable exciton radiative recombination probability, despite the different conjugation length and functionalisation.

**Figure 3:**
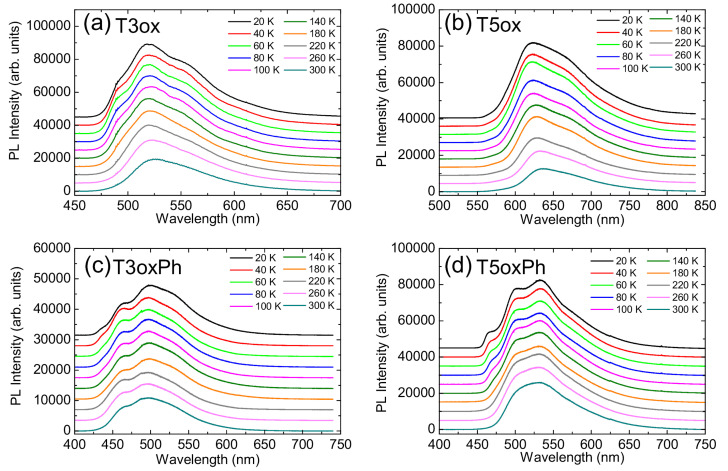
Temperature dependence of the PL spectra of T3ox (**a**), T5ox (**b**), T3oxPh (**c**), and T5oxPh (**d**). The spectra are vertically offset for clarity.

In order to have a deeper understanding of the non-radiative decay processes (and of their dependence on the molecular structure), the temperature dependence of the PL spectra was investigated (see Figure 3).

The PL spectra at low temperature can be reproduced, for all the molecules, by a superposition of equally spaced (in energy) peaks (see Figure S1). The peak-to peak-distance is compatible, for all the molecules, with the C-C in plane vibration energy (about 180 meV [57]), allowing one to ascribe the peak at the highest energy (lowest wavelength) to the S${}_{1}\to$ S${}_{0}$ 0-0 line and the other peaks to vibronic replicas.

For all molecules, as temperature increases, the spectra show no appreciable spectral shift, but do exhibit a broadening of the phonon replicas, which become less resolved.

In addition, a clear progressive intensity quenching is present (see Figure S2), evidencing a temperature-induced variation in the interplay between radiative and non-radiative recombination. The relative quenching between room and low temperature is higher in T3ox (about 2.3 times) and T5ox (about 3.5 times) than in the corresponding phenylated molecules (about 1.4 times in T3oxPh and 1.5 times in T5oxPh).

A first indication on the origin of the observed PL intensity temperature dependence can be obtained by a looking at the values of the relative room temperature/low temperature intensity quenching and of the room temperature PLQY. In particular, the PLQY is the ratio between the number of emitted photons and the number of pump laser absorbed photons; thus, assuming that the samples’ absorption does not significantly change in temperature, the PLQY and the integrated PL intensity are directly proportional:

(3) $$I_{PL}\propto PLQY⟺\frac{I_{PL}\left(T\right)}{PLQY\left(T\right)}=C=\frac{I_{PL300}}{PLQY_{300}}⟺PLQY\left(T\right)=\frac{I_{PL}\left(T\right)}{I_{PL300}}\cdot PLQY_{300}$$

where $I_{PL}\left(T\right)$ and $PLQY\left(T\right)$ are the PL intensity and PLQY at a generic temperature, respectively, C is the proportionality constant between PLQY and PL intensity, and $I_{PL300}$ and $PLQY_{300}$ are the PL intensity and PLQY values at room temperature. In other terms, the ratio between the integrated PL intensities at a generic and at room temperatures also quantifies the relative PLQY increase between the two temperatures. Looking at the PL intensity variation between low (T = 20 K) and room temperature, a PL intensity reduction of about 2.3, 1.4 and, 1.5 times for T3ox, T3oxPh, and T5oxPh, respectively, is observed, that, considering the room temperature PLQY values, allows one to estimate that the PLQY at low temperature is close to 100%. This result evidences the almost complete suppression of non-radiative relaxation channels at low temperature and strongly suggests that the PL quenching as the temperature increases mainly comes from a progressive decrease in the non-radiative lifetime. For T5ox, a much higher PL intensity relative quenching (about 3.5 times) is observed; however the room temperature PLQY is also largely the lowest, resulting in an estimated low temperature PLQY of about 42%, evidencing that the radiative and non-radiative times are almost comparable.

**Figure 4:**
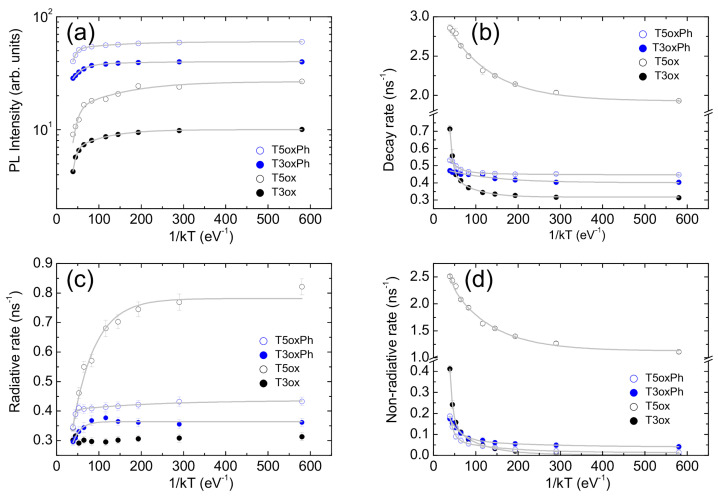
Temperature dependence of the PL intensity (**a**), total PL decay rate (**b**), radiative decay rate (**c**), and non-radiative decay rate (**d**). The grey lines are the best fit curves with Equations (4), (5) and (8) for PL intensity, total decay rate, and radiative and non-radiative decay rates, respectively.

In order to have quantitative insight into these features, the temperature dependence of the total PL intensity was analysed. The obtained values (see Figure 4a) show an almost constant value at low temperature, followed by an initial progressive weak decrease and then a stronger decrease as the temperature increases. These features are the typical signatures of the presence of two thermally activated non-radiative processes and allow one to adapt, in the minimum $\chi^{2}$ sense, the data to the following Arrhenius equation:

(4) $$I_{PL}\left(T\right)=\frac{I_{0}}{1+A_{1}e^{-\frac{\Delta E_{1PL}}{kT}}+A_{2}e^{-\frac{\Delta E_{2PL}}{kT}}}$$

where $I_{0}$ is the PL intensity at T = 0 K, *k* is the Boltzmann constant, $\Delta E_{1PL}$ and $\Delta E_{2PL}$ are the activation energies of the two PL quenching processes, and $A_{1}$ and $A_{2}$ are the corresponding coupling constants. The experimental data are well reproduced by the best fit line (see Figure 4a) for the activation energy values reported in Table 2.

For all the molecules, the activation energy of the first quenching process is on the order of about 10 meV, and the activation energy of the second process is between about 45–50 meV in T5ox, T3oxPh, and T5oxPh and about 150 meV in T3ox.

In order to better understand the origin of the observed PL intensity temperature dependence and the nature of the thermally activated processes, the PL relaxation dynamics as a function of the temperature were also measured.

**Figure 5:**
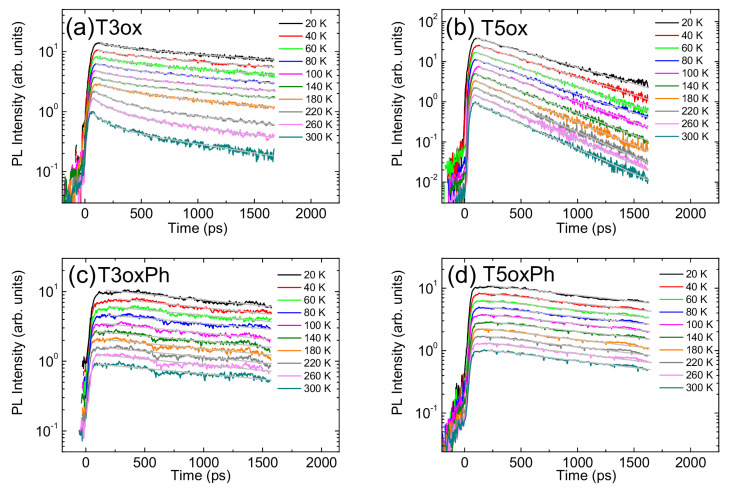
Temperature dependence of the PL relaxation dynamics of T3ox (**a**), T5ox (**b**), T3oxPh (**c**), and T5oxPh (**d**). The grey lines are the best fit curves with an exponential decay. The signals are normalised to the peak value and scaled for clarity.

In T3ox (see Figure 5a), an almost mono-exponential decay is observed at low temperature, with a lifetime of about 3 ns, and, as the temperature increases, the appearance of a second faster relaxation process with a lifetime of about 0.2 ns emerges, with a progressively increasing relative contribution to the PL relaxation. This behaviour, and the time scale of the fast process, allows to ascribe this process to the thermal activation of energy migration toward non-radiative relaxation sites [58] and the slower process to exciton recombination. As the temperature increases, the relaxation becomes progressively faster [59].

In all the other molecules, a mono-exponential PL relaxation is observed at all the temperatures, becoming progressively faster as the temperature increases (see Figure 5b–d). One can also observe that the relative lifetime decrease with the temperatures is much more evident for T3ox and T5ox than for the corresponding phenylated molecules.

The observed progressive lifetime decrease as the temperature increases is qualitatively consistent with the previously suggested presence of thermally activated non-radiative decay channels, decreasing the non-radiative decay time—and, thus, the total decay time—and causing a PL intensity quenching. As the relaxation probability is directly quantified by the decay rate (k=1/τ), the temperature dependence of the relaxation dynamics was quantitatively analysed in terms of total decay rate instead of total decay lifetime. Assuming a temperature-independent radiative rate ($k_{r}$) and two thermally activated non-radiative processes (as evidenced by the PL quenching analysis), the total rate can be written as follows:

(5) $$k\left(T\right)=k_{r}+k_{nr0}+k_{anr1}e^{-\frac{\Delta E_{nr1}}{kT}}+k_{anr2}e^{-\frac{\Delta E_{nr2}}{kT}}$$

where $k_{0nr}$ is the non-radiative rate at T = 0 K, $\Delta E_{nr1}$ and $\Delta E_{nr2}$ are the activation energies of the two thermally activated non-radiative processes, and k${}_{anr1}$ and k${}_{anr2}$ are the corresponding coupling constants. From this expression, one immediately obtains Equation (4) as an expression of the PL intensity temperature dependence.

For all the molecules, the best fit functions excellently reproduce the experimental data (see Figure 4b) for the activation energies best fit values reported in Table 2.

For T3ox the best fit activation energies are in excellent agreement with the values obtained from the PL intensity quenching analysis, allowing to ascribe the temperature dependence of both the PL intensity and the total decay rate to the thermal activation of two non-radiative processes.

On the contrary, and rather unexpectedly, for the other three molecules, only one thermally activated process is observed, with an activation energy consistent with $\Delta E_{1PL}$ in T3oxPh and T5ox and with $\Delta E_{2PL}$ in T5oxPh. Such a disagreement clearly demonstrates the inadequacy of the used models to correctly describe both the PL intensity and the total decay rate temperature dependence and, thus, that the experimental results cannot be only ascribed to the presence of thermally activated non-radiative processes.

The analysis was thus improved by exploiting again the possibility to separate the radiative and non-radiative contribution to the decay rate by knowing the lifetime and the PLQY, as well as the proportionality between PLQY and PL intensity:

(6) $$\begin{array}{ccc} & & PLQY\left(T\right)=\frac{\tau \left(T\right)}{\tau_{r}\left(T\right)}=\frac{k_{r}\left(T\right)}{k\left(T\right)}⟺k_{r}\left(T\right)=\frac{PLQY\left(T\right)}{\tau \left(T\right)} \end{array}$$

(7) $$\begin{array}{ccc} & & k_{nr}\left(T\right)=k\left(T\right)-k_{r}\left(T\right) \end{array}$$

where k${}_{r}=1/\tau_{r}$ and k${}_{nr}=1/\tau_{nr}$ are the radiative and non-radiative decay rates, respectively.

The obtained decay rate values (see Figure 4c,d) allow one to observe several interesting features. In T3ox, the radiative rate is independent of the temperature, while the non-radiative rate is almost zero at low temperature and it progressively increases with the temperature. These results are fully consistent with the previous conclusions and, thus, confirm that, in this molecule, the PL intensity quenching is fully due to the thermal activation of non-radiative decay processes.

On the contrary, the other three molecules show not only a progressive increase in the non-radiative rate as the temperature increases, but also a clear progressive decrease in the radiative rate, which is very strong in T5ox (about 2.3 times) and rather weak in T3oxPh and T5oxPh (about 25%). It is also interesting to observe that, even if the molecules show comparable radiative decay rate (time) at room temperature (also observed in solution and in theoretical predictions on molecules with optimised geometry [41]), suggesting that the chemical structure mainly affects the non-radiative rate (time), the radiative rates at low temperature are clearly different.The comparable radiative rates at room temperature thus come from the combination of different low temperature values and a different entity of the temperature-induced variation.

It can be further observed that, rather counter-intuitively, the molecule with the highest low temperature radiative rate is T5ox, that is, the molecule showing the lowest PLQY at room temperature and also the lowest estimated PLQY at low temperature. However, T5ox also shows the highest non-radiative rate (explaining the lower estimated PLQY at low temperature) and the strongest radiative reduction from low to room temperature, which, overall, leads to the lowest PLQY at room temperature.

Moving to the analysis of the radiative and non-radiative rates’ temperature dependence, a near-constant value is observed at low temperature, and a progressively increasing variation is observed as the temperature increases, again evidencing the presence of thermally activated processes. Adapting Equation (5) to radiative and non radiative rates one now has the following:

(8) $$k_{i}\left(T\right)=k_{0i}+k_{ai1}e^{-\frac{\Delta E_{i1}}{kT}}+k_{ai2}e^{-\frac{\Delta E_{i2}}{kT}}$$

where *i* = *r*, *nr*, $k_{0i}$ is the rate value at *T* = 0 K, $\Delta E_{i1}$ and $\Delta E_{i2}$ are the activation energies of the thermally activated non-radiative processes, and $k_{ai1}$ and $k_{ai2}$ are the corresponding coupling constants.

For all the molecules, the best-fit curves excellently reproduce (see Figure 4c,d) the experimental values of both the radiative and non-radiative rates for the best fit values of the activation energies and the coupling constants reported in Table 2 and Table S1, respectively.

The obtained values now allow one to fully rationalise the observed inconsistencies between the total decay rate and PL intensity activation energies and to correctly determine the origin (radiative or non-radiative) of the observed PL intensity dependence.

A straightforward behaviour is found in T3ox, which does not show any temperature dependence of the radiative decay rate and also shows fully consistent activation energies of the PL quenching, the total decay rate, and the non-radiative decay rate. In this molecule, the observed temperature-induced PL quenching can be, thus, fully ascribed to the thermal activation of non-radiative decay channels.

Moving to T5ox, the discrepancy between the total lifetime and PL quenching activation energies is due to the presence of a combined temperature induced radiative rate decrease and non-radiative rate increase. In particular, the two activation energies of the radiative rate decrease are comparable to activation energies of the non-radiative rate increase. However, looking at the coupling constants’ best fit values (see Table S1), it can be observed that the total contributions of the two effects basically cancel each other out for the process with the higher activation energy (see Supporting Information for more details), thus leading to a total rate temperature dependence that only shows a progressive decrease, with an activation energy consistent with the lowest activation energy. On the contrary, when looking at the PL intensity, both the radiative rate decrease and the non-radiative rate increase result in PL quenching, leading to the presence of low and high activation energies, comparable to the corresponding low and high activation energies of the radiative and non-radiative rates’ variation. The same kind of analysis also allows one to explain the presence of only one thermally activated process visible in the total rate, while two processes are observed for the radiative and non-radiative rates as well as for the PL quenching (see Supporting Information for more details).

A similar analysis allows one to ascribe the PL quenching activation energies to radiative or non-radiative effects (see Table 2). As already observed, in T3ox, the PL quenching with temperature can be fully ascribed to the thermal activation of non-radiative decay processes.

In T5ox and in T5oxPh, the PL quenching activation energies are similar to the corresponding activation energies of the radiative rate decrease and non-radiative rate increase. Thus, in these molecules, the observed PL quenching comes from an interplay between the decrease in the radiative rate and the decrease in the non-radiative one.

Finally, in T3oxPh, the quenching process with lower activation energy can be ascribed to the thermal activation of the non-radiative rate increase with the corresponding activation energy, while the quenching with higher activation energy again comes from a combination of radiative and non-radiative effects.

The origin of the processes involved in the radiative and non-radiative variation in temperature can be determined by looking at the obtained values of the corresponding activation energies. In particular, up to about 180 K, the temperature dependence in mainly due to the process with activation energy $\Delta E_{1}$, showing values between a few meV and 15 meV, which is the typical range of vibration energies associated with inter-ring libration modes in standard thiophenes [60], oligothiophene-S,S-dioxides [61], and oligophenylenes [62].

It can, thus, be concluded that, in all the molecules, the thermal activation of low energy vibrations, related to inter-ring libration of the lateral rings [63], increases the non-radiative rate and determines a PL quenching.

The presence of a small radiative rate increase in T5ox and T5oxPh with activation energy $\Delta E_{1}$ suggests a contribution of these vibrations also to a small decrease in the HOMO–LUMO wavefunctions’ overlap. The lack of a similar behaviour in the shorter T3ox and T3oxPh could indicate that the effect is mainly due to the libration of the two external rings and/or that it is observable only in the pentamers, due to the higher wavefunction delocalisation in the longer molecules.

At temperatures above 180 K, the temperature variations are instead dominated by the process with activation energy $\Delta E_{2}$ that, in T3oxPh, T5ox, and T5oxPh, is on the order of 40–50 meV. In oligothiophenes, this is the characteristic range of S-C in-plane and out-of-plane torsional modes, and of the C-C in-plane torsional mode [64], while, in oligophenylenes, this corresponds to the energy of out-of-plane C-C stretching [65]. The thermal activation of these vibrations also determines the decrease in the radiative rate, which is particularly strong for T5ox.

The only molecule with a different behaviour is T3ox, which shows a much higher $\Delta E_{2}$ value, in the typical range of C-C inter-ring vibrations [57], and no variations in the radiative rate. A possible origin of this peculiar behaviour can be the highly distorted geometrical structure of the molecules in crystalline powders [45], which probably prevents efficient torsional vibrations, allowing one to observe the effects of the thermal activation of high energy vibrations.

As a last point, the performed analysis also allows one to understand the quantitative differences in the relative quenching between room and low temperature of molecules with lateral thiophene rings and the phenylated molecules, i.e., the factor that mainly determines the different PLQY at room temperatures. In particular, even if the activation energies of the quenching processes are basically comparable between molecules with lateral phenyl and thiophene rings, the phenylated molecules always clearly show lower values of the corresponding coupling constants (see Table S1). This result evidences a lower probability of decay rate variation (for a given activation energy), and it is, thus, a signature of higher structural rigidity of the molecule [41,66,67].

Overall, these results demonstrate that the PLQY in this class of molecules is mainly affected by the thermal activation of intermolecular vibrations, confirming that, also in the solid state, the main non-radiative process is IC instead of ISC, as suggested by experiments in solution [29,41,46], evidencing that the higher PLQY values in phenylated molecules come from a higher molecular rigidity. In addition, the similarity of the behaviour of the molecules in the solid state with the ones isolated in solutions clearly demonstrates that the emission properties of functionalised dioxide-oligothiophenes are mainly determined by the intramolecular properties, with no detrimental effects related to intermolecular interactions.

Partially different from this behaviour is T5ox, which clearly shows a lower PLQY with respect to the other molecules. This lower PLQY can be ascribed to the higher length with respect to T3ox, that reduces the packing effects related to the polar SO${}_{2}$ group and leads to a packing resembling the cofacial packing of standard oligothiophenes, as also previously reported in the literature [45]. A further contribution to the lower PLQY of T5ox comes from the increase in the IC when the emission wavelength increases [68], leading to a more efficient IC in T5ox that emits in the red regon, as also clearly demonstrated by the much higher coupling constants of the decay rate variations.

Finally, this analysis demonstrates that the much higher PLQY in the solid state than in solution, and, thus, the AIEgens behaviour of oligothiophene-S,S-dioxides, primarily comes from a much higher rigidity of the environment surrounding the molecules in the solid state.

## 3. Conclusions

In conclusion, the origin of the high PLQY in the solid state of in different oligothiophene-S,S-dioxides was investigated by temperature-dependent PL and time resolved PL measurements. It was demonstrated that the room temperature emission properties of the molecules are determined by an interplay between the intrinsic intramolecular properties and the thermal activation of intramolecular vibrations. In particular, both the radiative and non-radiative decay rates are affected by the thermal activation of vibrations with quantitative differences between different molecules related to their molecular structure. The higher PLQY of molecules with lateral phenyl rings, observed also in other functionalised thienyl-S,S-dioxide oligomers [35], is mainly related to weaker coupling constants with the vibrations and, thus, to a higher molecular rigidity.

These results demonstrate the potentiality of temperature dependent spectroscopy to investigate the basic photophysics of active molecules, and are expected to suggest future chemical strategies to develop molecules with high PLQY in the solid phase.

## 4. Materials and Methods

The molecules have been synthesised as described in Ref. [35] and investigated in the form of microcystalline powder (sandwiched between two quartz substrates).

The PLQY measurements have been performed by exciting the films inside an integrating sphere [69], with a CW He–Cd laser (λ = 325 nm) and collecting the sample emission with an Ocean Optic 2000 spectrometer, with a spectral resolution of 4 nm. The typical statistical uncertainty of the measurement is about 3% of the obtained value.

The PL and time resolved PL experiments were performed by exciting the samples using the second harmonic (λ = 390 nm) of a Spectra Physics Tsunami Ti-Sapphire laser, delivering 2 ps pulses with a repetition rate of 82 MHz.

The sample emissions were collected with a telescopic lens system and dispersed, for the PL experiments, by a TRIAX 320 spectrometer (spectral resolution 0.8 nm) coupled with a Si-CCD, and, for the time resolved measurements, by a spectrometer coupled to a streak camera (temporal resolution 10 ps). The sample temperature was changed by a closed circle He cryostat, in the range from 20 K to 300 K.

## Figures and Tables

**Table 1:** Room temperature PL decay time, PLQY, and radiative and non-radiative lifetimes for the investigated molecules.

Molecule	Decay Time (ns)	PLQY (%)	Radiative Time (ns)	Non-Radiative Time (ns)
T3ox	1.65 ± 0.05	46.0 ± 1.4	3.60 ± 0.18	3.10 ± 0.18
T3oxPh	2.13 ± 0.05	63 ± 2	3.38 ± 0.14	5.8 ± 0.5
T5ox	0.350 ± 0.010	12.0 ± 0.4	2.92 ± 0.13	0.398 ± 0.013
T5oxPh	1.88 ± 0.02	70 ± 2	2.90 ± 0.11	5.4 ± 0.3

**Table 2:** PL quenching, total decay rate, and radiative and non-radiative rate best fit activation energies for all investigated molecules.

Molecule	ΔE${}_{1PL}$ (meV)	ΔE${}_{2PL}$ (meV)	ΔE${}_{1k}$ (meV)	ΔE${}_{2k}$ (meV)	ΔE${}_{1r}$ (meV)	ΔE${}_{2r}$ (meV)	ΔE${}_{1nr}$ (meV)	ΔE${}_{2nr}$ (meV)
T3ox	14.7 ± 1.6	148 ± 21	19 ± 4	150 ± 30	-	-	15.0 ± 1.3	145 ± 11
T3oxPh	9 ± 3	48 ± 6	8.9 ± 1.6	-	55 ± 13	-	10 ± 2	39 ± 6
T5ox	7 ± 2	45 ± 10	9.5 ± 0.3	-	6 ± 2	36 ± 5	8 ± 2	34 ± 6
T5oxPh	8.5 ± 1.2	50 ± 6	39 ± 5	-	1.5 ± 0.4	35 ± 4	8.2 ± 1.1	39 ± 6

## Data Availability

The data can be obtained from the authors in case of reasonable request.

## Supplementary Materials

The following supporting information can be downloaded at: https://www.mdpi.com/article/10.3390/molecules28135161/s1, Figure S1: PL spectra of the investigated molecules at T = 20 K (black lines). The red lines are the best fit curves with a multigaussian fit function and the green lines the individual peaks. The arrow evidences the spacing expected for C-C stretching vibronic replicas; Figure S2: Temperature dependence of the PL spectra of the investigated molecules, without vertical shift in order to evidence the progressive intensity quenching. Only few temperatures are shows for clarity. Table S1: best fit values of the coupling constants of the thermally activated processes in the total, radiative and non-radiative rate temperature dependence.
