# Supplementary material for: Investigation of the Origin of High Photoluminescence Quantum Yield in Thienyl-S,S-dioxide AIEgens Oligomers by Temperature Dependent Optical Spectroscopy
Source: Molecules. 2023 Jul 1;28(13):5161. doi: 10.3390/molecules28135161 (PMC10343292; doi:10.3390/molecules28135161)
Supplement: Supplementary file 1 [file molecules-28-05161-s001.zip › molecules-2473954-supplementary.pdf]

Supporting Information

Investigation of the Origin of High Photoluminescence Quantum Yield in Thienyl-S,S-dioxide AIEgens Oligomers by Temperature Dependent Optical Spectroscopy

Marco Anni

Dipartimento di Matematica e Fisica "Ennio De Giorgi", Università del Salento, Via per Arnesano, 73100 Lecce, Italy; marco.anni@unisalento.it

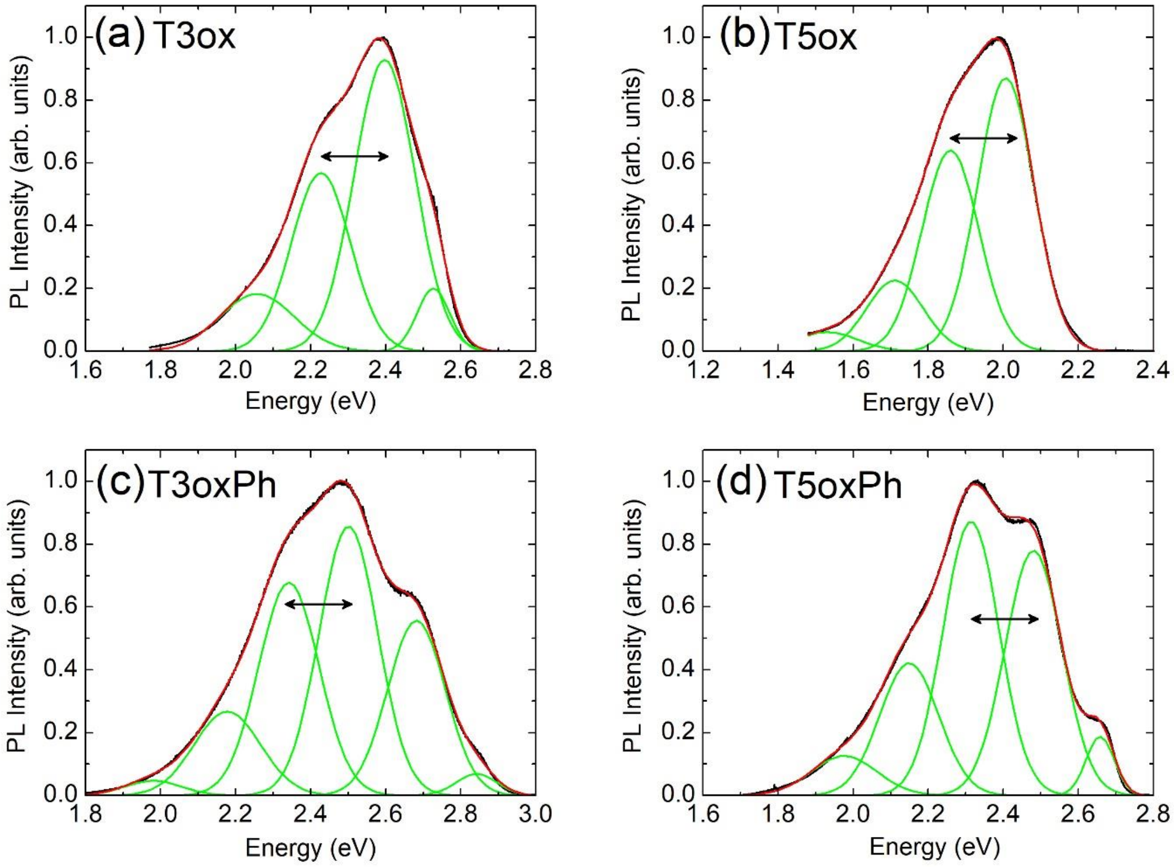

Figure S1: PL spectra of the investigated molecules at T=20 K (black lines). The red lines are the best fit curves with a multigaussian fit function and the green lines the individual peaks. The arrow evidences the spacing expected for C-C stretching vibronic replicas.

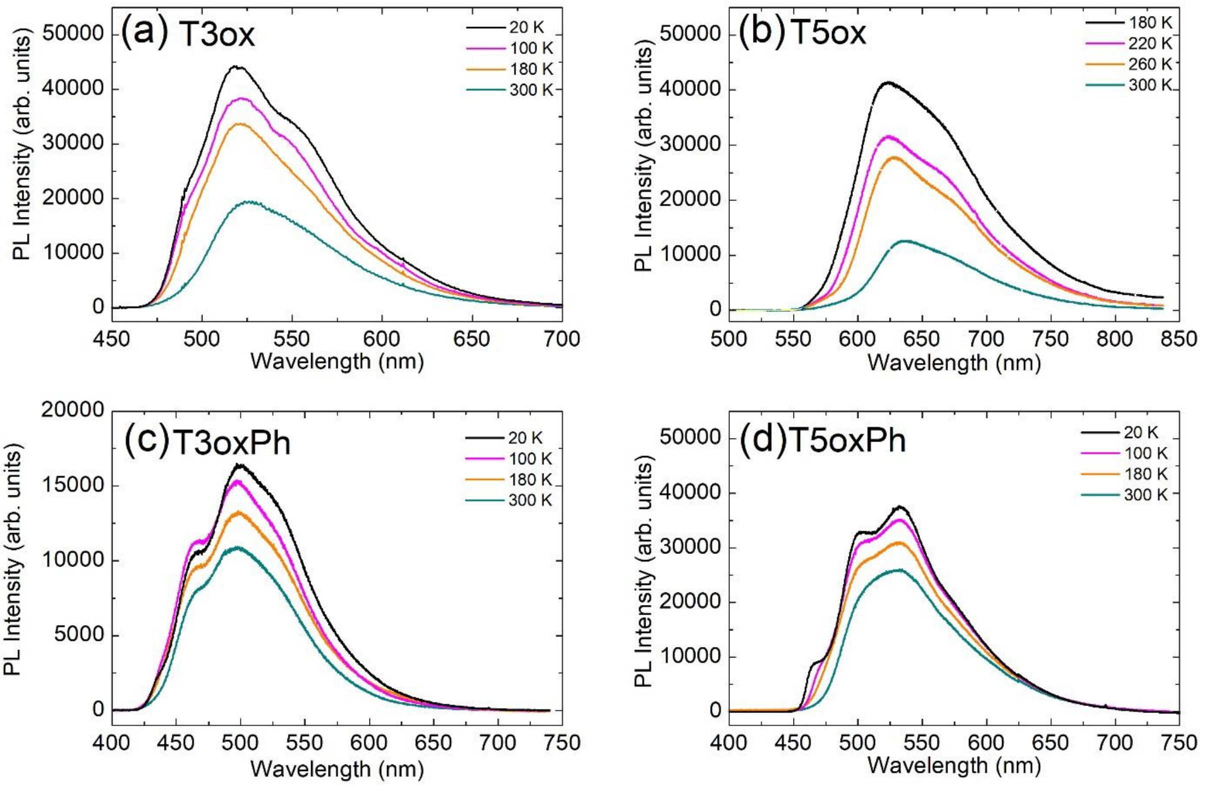

Figure S2: Temperature dependence of the PL spectra of the investigated molecules, without vertical shift in order to evidence the progressive intensity quenching. Only few temperatures are shows for clarity.

Radiative, non-radiative and total rate temperature dependence

In this section we quantitatively determine the expression of the total decay rate, considering that both the radiative and non-radiative rates are temperature dependent, in order to explain why the activation energies of the total rate and of the PL quenching can be different.

Remembering that the total rate is simply the sum of the radiative and non-radiative ones, and using Equation 5 for the temperature dependence of both rates we have:

$$k(T) = k_r(T) + \ k_{nr}(T) =$$

$$= k_{0r} + k_{ar1}e^{-\frac{\Delta E_{1r}}{kT}} + k_{ar2}e^{-\frac{\Delta E_{2r}}{kT}} + k_{0nr} + k_{anr1}e^{-\frac{\Delta E_{1nr}}{kT}} + k_{anr2}e^{-\frac{\Delta E_{2nr}}{kT}} \qquad \text{(S1)}$$

From this equation we can now determine the total rate temperature dependence of all the molecules.

Starting from T3ox we observe that this molecule shows a temperature independent radiative decay rate. The temperature dependence of the radiative rate thus only include the constant term and Equation S1 becomes:

$$k(T) = k_{0r} + k_{0nr} + k_{anr1}e^{-\frac{\Delta E_{1nr}}{kT}} + k_{anr2}e^{-\frac{\Delta E_{2nr}}{kT}}$$

With the substitution $k_{0r} + k_{0nr} = k_0$ the equation becomes identical to Equation 3.

Thus, the activation energies of the total and of the non-radiative rate are the same and also coincide with the PL quenching ones, as actually observed.

Moving to T5ox, the radiative rate shows the same activation energies of the non-radiative rate, thus $\Delta E_{1r} = \Delta E_{1nr}$ and $\Delta E_{2r} = \Delta E_{2nr}$ and Equation S1 becomes:

$$k(T) = k_{0r} + k_{ar1}e^{-\frac{\Delta E_{1r}}{kT}} + k_{ar2}e^{-\frac{\Delta E_{2r}}{kT}} + k_{0nr} + k_{anr1}e^{-\frac{\Delta E_{1nr}}{kT}} + k_{anr2}e^{-\frac{\Delta E_{2nr}}{kT}} =$$

$$= k_{0r} + k_{0nr} + (k_{ar1} + k_{anr1})e^{-\frac{\Delta E_{1r}}{kT}} + (k_{ar2} + k_{anr2})e^{-\frac{\Delta E_{2r}}{kT}}$$

Looking at the best fit values of k_{ar2} and k_{anr2} (see Table S1) we observe that $k_{ar2} + k_{anr2} \approx 0$, thus the previous equation just becomes:

$$k(T) = k_{0r} + k_{0nr} + (k_{ar1} + k_{anr1})e^{-\frac{\Delta E_{1r}}{kT}}$$

The total rate thus only shows a variation due to one of the two thermally activated non-radiative processes and $\Delta E_{1nr} = \Delta E_{1k}$, as observed.

A very similar situation is shown by T3oxPh, with a compensation between the radiative rate decrease and the contribution to the non-radiative rate increase of the process with activation energy ΔE_{2nr}, leading to a total rate decrease basically due to the non-radiative process with activation energy ΔE_{1nr}.

Concerning T5oxPh we have $\Delta E_{1r} \approx \Delta E_{1nr}$ and $k_{ar1} + k_{anr1} \approx 0$, leading to the absence of a term with activation energy ΔE_1in the total rate. The second activation energy is also similar for the radiative and non-radiative rates, but the non-radiative coupling constant is higher than the radiative one, determining a decrease of the total rate with coupling constant $k_{a1} = k_{ar2} + k_{anr2}$.

Molecule	k_{a1} (ns^{-1})	k_{a2} (ns^{-1})	k_{ar1} (ns^{-1})	k_{ar2} (ns^{-1})	k_{anr1} (ns^{-1})	k_{anr2} (ns^{-1})
T3ox	$(2.9\pm0.8)10^{-1}$	$(7.8\pm0.8)\ 10^{1}$	-	-	$(2.6\pm0.3)10^{-1}$	$(7\pm1)10^{1}$
T5ox	1.31 ± 0.03	-	$-(2.9\pm0.6)10^{-1}$	-1.1 ± 0.2	1.30 ± 0.13	1.6 ± 0.3
T3oxPh	$(9.8\pm0.9)10^{-2}$	-	$-(6\pm1)10^{-1}$	-	$(6.9\pm1.3)10^{-2}$	$(4.5\pm0.6)10^{-1}$
T5oxPh	$(3.8\pm0.8)10^{-1}$	-	$-(5.4\pm0.6)10^{-2}$	$-(4.9\pm0.6)10^{-1}$	$(8\pm1)10^{-2}$	$(9.0\pm1.6)10^{-1}$

Table S1: best fit values of the coupling constants of the thermally activated processes in the total, radiative and non-radiative rate temperature dependence.
